# Idiopathic sclerosing mesenteritis in paediatrics: Report of a successfully treated case and a review of literature

**DOI:** 10.1186/1546-0096-8-5

**Published:** 2010-01-21

**Authors:** Vijay Viswanathan, Kevin J Murray

**Affiliations:** 1 Department of Pediatric Rheumatology, Princess Margaret Hospital for Children, Perth, WA, Australia

## Abstract

A 6 year old female with symptoms of small bowel obstruction underwent an exploratory laparotomy which revealed widespread evidence of inflammatory fibrotic adhesions involving the jejunal mesentery. In view of persistent growth failure, chronic anaemia, elevated acute phase reactants and imaging evidence of a diffuse progressive inflammatory process, the child was treated with corticosteroids and methotrexate with complete response. The literature on juvenile idiopathic sclerosing mesenteritis has been reviewed.

## Background

Idiopathic sclerosing mesenteritis (ISM) is an uncommon disease involving the small bowel mesentery and characterised by chronic inflammatory changes ultimately progressing to fibrosis. The first reported series featured 34 cases of "retractile mesenteritis and mesenteric sclerosis"; published in 1924 [1-3]. It has been described variously as fibrosing mesenteritis, retractile mesenteritis, liposclerotic mesenteritis, xanthogranulomatous mesenteritis, mesenteric Weber Christian disease and systemic nodular panniculitis [4]. These names possibly reflect the underlying pathology involved with mesenteric lipodystrophy (predominant fatty degeneration), mesenteric panniculitis (chronic inflammation) and retractile mesenteritis (predominant fibrosis) being the common histologic variants. It has been postulated that these variants represent the varied spectrum of a single disease process which could be termed idiopathic sclerosing mesenteritis [5].

Though the exact aetiology remains obscure, proposed mechanisms include prior abdominal trauma/surgery, autoimmunity, infection and ischemia [6]. A wide range of therapeutic initiatives have been reported including corticosteroids, colchicine, immunosuppressives (cyclophosphamide and azathioprine), tamoxifen, thalidomide and hormonal therapies with varying success [6,7]. The disease is considered rare in the pediatric age group with only 16 cases reported to date. We describe a case of ISM in a young girl who was treated successfully with corticosteroids and methotrexate, and briefly review the pediatric literature on this subject.

## Case presentation

A 6 year old girl presented at a local hospital with an acute onset of abdominal pain, nausea and bilious vomiting. A history of constipation, recurrent vomiting associated with high fever in the preceding 12 months was elicited. Clinical examination was reported to be normal at the time. Complete blood counts revealed haemoglobin (Hb) 94 g/l, WBC 18.4 × 0^9^/l (Absolute PMN 15.6 × 10^9^/l, Lymph 2.0 × 10^9 ^/l) and platelets 663 × 10^9 ^/l. Her ESR was elevated at 54 mm/hr [1-15 mm/hr) and CRP 84 mg/l (<10 mg/l). Ultrasonography of the abdomen revealed diffuse, mesenteric lymphadenopathy. A barium meal with follow through suggested a small bowel obstruction. A laparotomy showed extensive intraperitoneal adhesions, mesenteric lymphadenopathy and a tight jejunal band with obstruction. The adhesions were divided, the obstruction was relieved and the patient's bowel function recovered steadily. Tissue cultures were negative. Histopathological analysis of the peritoneal tissue revealed fibro-fatty tissue with extensive fibrosis throughout, infiltrated by many scattered lymphocytes, plasma cells and occasional neutrophils but no malignant cells. The single lymph node examined revealed dilated sub-capsular sinuses with an expanded para-cortex with mixed histiocytes and occasional immunoblast with no granulomas, Reed Sternberg cells or any evidence of lymphoma. Ziehl-Neelsen stains were negative for tuberculosis organisms as was subsequent culture. The clinical condition improved slowly over the subsequent 2 weeks and she was discharged on oral iron supplements. No evidence of mono-clonality was seen on flow cytometry for immuno-phenotyping of lymph node tissue.

**Figure 1:**
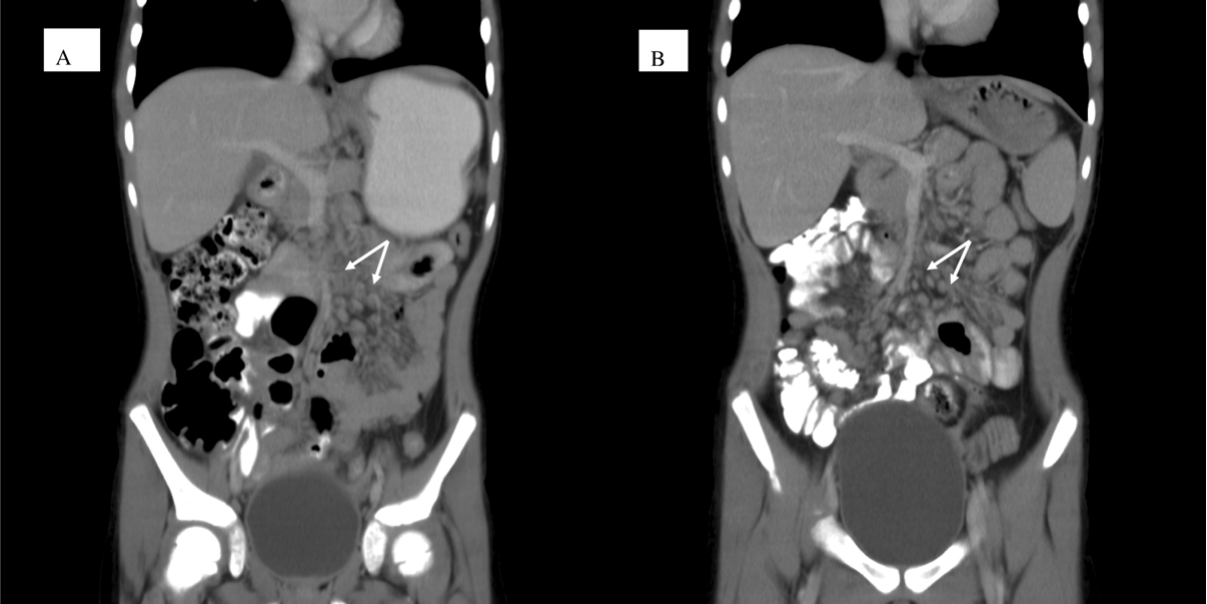
**A (Pre treatment) Radiological findings demonstrating altered attenuation of mesentery and diffuse lymphadenopathy (white arrows)**. **B **(Post treatment) Resolution of lymphadenopathy.

Three months later, she moved interstate and presented to the gastroenterology clinic at our hospital with continued weight loss and an exacerbation of abdominal pain and vomiting of fecal matter. Upper and lower endoscopies were normal. MRI of the abdomen revealed apparent thickening of a loop of proximal jejunum in the left mid abdomen with enlarged mesenteric lymph nodes. CT scan revealed evidence of a subtle attenuation in the mesentery with diffuse lymphadenopathy (Figure 1A). The symptoms of obstruction settled with conservative management. At one month follow up, her haemoglobin remained depressed, 87 g/l, WBC 15.0 × 10^9^/l with thrombocytosis. She had lost 7 kgs in weight (from the first presentation), had recurrent abdominal pain and required a second admission for a partial obstruction which resolved with conservative management.

A rheumatology consultation was made. Review of the case revealed multiple episodes of subacute small bowel obstruction with chronic anaemia, weight loss, and a chronic inflammatory process with fibrosis on mesenteric biopsy and elevated acute phase reactants. Further investigations revealed a borderline positive ANA of 1:40 (speckled pattern), a negative dSDNA and rheumatoid factor (RF). Total serum proteins were 82 g/l, albumin 40 g/l (32-48 g/l), globulins 42 g/l (23-35 g/l), and serum IgG 15.7 g/l (6-12.3 g/L) indicating mild hypergammaglobulinemia. IgG subclasses IgG1 11.1(4.0-10.8 g/L), IgG2 4.3 (0.8-4.1 g/L), IgG 30.4 (0.1-1.4 g/L), IgG4 1.47 (<1.9 g/L) were unremarkable. An abdominal angiogram was done to screen for major vessel vasculitis such as polyarteritis nodosa, and was negative.

**Figure 2:**
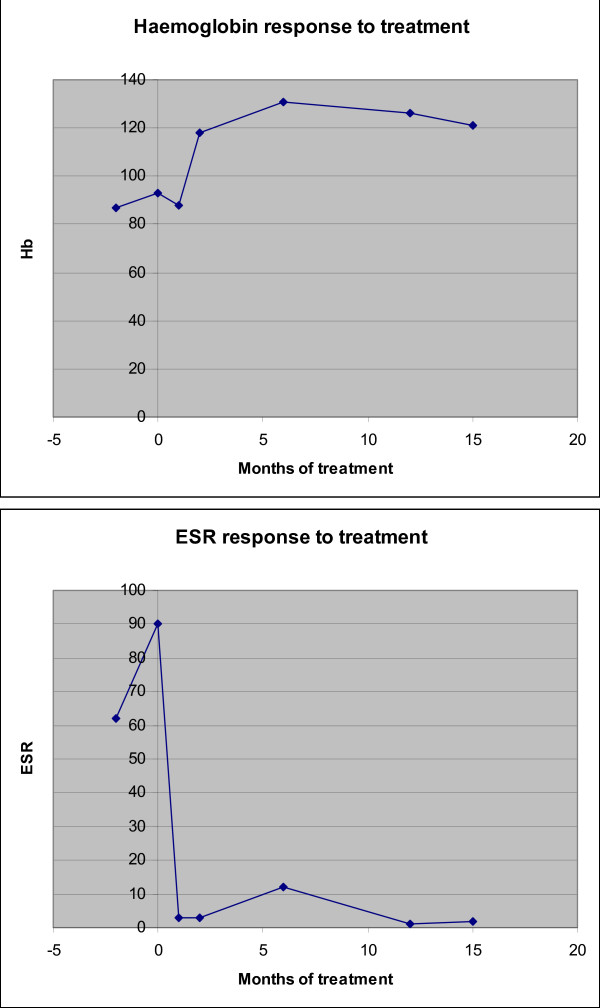
**Response to treatment of patients ESR and Haemoglobin in month since the start of treatment**.

A diagnosis of idiopathic sclerosing mesenteritis was made and the child was treated with 3 daily pulses of methyl prednisolone therapy repeated monthly for 3 months. She made a remarkable and rapid clinical recovery with marked reduction in fatigue, abdominal pain and resolution of fever within 2 weeks. Four weeks into treatment, laboratory investigations revealed significant improvement; Hb 118 g/l, WBC 10.1 × 10^9^/l platelets 490 × 10^9^/l and ESR 3 mm/hr. Subcutaneous methotrexate (10 mg/m^2 ^), total dose 10 mg once a week was started for long term immunomodulation and steroid sparing effects. At 6 months follow - up from the onset of treatment, she had gained 3 kgs, was completely asymptomatic and her laboratory investigations remained normal. Her repeat CT scan this time had revealed resolution of the lymphadenopathy with normal mesentery (Figure 1b). Currently at 18 months follow up, she is under regular 3 monthly follow-up, off steroids and only on subcutaneous methotrexate at (10 mg/m^2) ^now 12.5 mg. Figure 2 illustrates the impressive and sustained response to treatment evidenced by her haemoglobin and ESR results over time.

## Discussion

First reported in 1924 as 'retractile sclerosing mesenteritis', ISM was reprised in a case series which reported it to be a benign disease in general. ISM is considered a rare disorder of late adult life with relatively few cases reported in world literature [1-3]. Only 3 major case series have been reported in adults comprising 53, 84 and 92 patients [5,6,8]. To our knowledge, only 17 pediatric cases including our own have been reported (see Table 1), with no gender predominance and age at diagnosis averaging 6.5 years (range 18 months -12 years).

**Table 1:** Clinical manifestations and course of illness in 17 children with sclerosing mesenteritis

Pt. no	Author/year	Age/Sex	Presenting features/duration	Treatment	Outcome	Special features (if any)
1	McGee et.al [18](1965)	6/M	A, Asc/unknown duration	Sx	Died ,3 weeks post surgery (Bacterial endocarditis )	No 'persistent fibrosis on autopsy.'

2	Soergel[16]et.al (1966)	14/M	A, WL, F/Recurrent symptoms over 12 yrs	Sx	Died ,12 yrs after initial symptom	Steatorrhea, secondary amyloidosis

3	Black et.al [19](1968)	8/F	A, N, V, D, F, M/6 weeks	Sx, Partial resection and ileocolostomy	UR/NF ,5 mths	Blunt trauma one week prior.

4	Spark et.al[17](1971)	6/M	A/M Two weeks	Sx/Hemi colectomy with ileo colic anastomosis	UR/NF ,9 mths	----------

5	Misaka et.al [20](1977)	4/F	A/C/V/Asc	Sx/enterostomy with resection	UR/NF ,12 mths	-----------

6	Melo et.al [21](1980)	13/F	A, N, V, M	Sx/hemicolectomy	NR	----------

7	Dor et.al (1982)	10/F	F	Sx/enterectomy, partial splenectomy and partial pancreatectomy	NR	SLE

8	Cakmak ^22^et. al (1986)	4/M	A/C/F/V, acute onset	Sx/Transverse laparotomy	UR/NF, 20 mths.	Recurrence after 2 weeks.

9	Jona et.al [23](1987)	5/F	A, F/7 days	Sx/Right hemicolectomy	UR/NF ,51/2 yrs	Bacterial peritonitis.

10	Davis et.al ^24^(1992)	3/M	A, An, V/one day	Sx/No resection.	UR/NF 2 mths.	Hemo Peritoneum

11	Ueda et.al [25](1997)	12/F	V, M/2 days	SRAM	UR/NF ,6 months	Fulminant hep A with recurrent ascites

12	Ito [20]et.al (1998)	8/M	NR	SRAM	UR/NF, 9 years	

13	Hakguder et.al ^11^(2000)	4/M	A, N, An/Not specified	SRAM	UR/NF, 6 months.	Meckels diverticulum

14	Kawano [20]et.al (2003)	2/M	F/1 month	SRAM	UR/NF	---------

15	Kawano [20]et.al (2003)	4/M	F, M/Not specified,	SRAM	UR/NF	-----------

16	Kawano [20]et.al (2003)	6/F	F, M/Not specified	SRAM	UR/NF	----------

17	Our case (2008)	6/F	A, V, WL, C/Recurrent episodes 6 months	Sx, Steroids, Methotrexate	UR/NF, 6 months.	Methotrexate first reported pediatric case.

Pt; Patient, A; Abdominal pain, An; Anorexia, Asc; Ascites, W; Weight loss, F; Fevers, M; Mass N/V; Nausea/Vomiting, C; Constipation, D; Diarrhoeas, Mtx; Methotrexate, St; Steroids, Sx; Surgery, SRAM; Surgical resection and anastomosis, UR; Uneventful recovery, NF; Normal follow up, NR; Not reported.

Various patho-physiological mechanisms suggested include previous surgery, trauma, hypoxia, allergy, infection and autoimmunity [6,9,10]. Concurrent intra abdominal pathologies have been reported in adults including lymphoma and ovarian tumours. One child had a coincidental presence of a Meckels diverticulum [11] while another had a blunt trauma to the abdomen a week before developing clinical manifestations. (See table 1). Other associated immuno-hematological anomalies such as common variable immunodeficiency, polyclonal gammopathies, acute myeloid leukaemia and myelodysplasias have been reported in adults. Understandably monoclonal/oligoclonal B cell expansion has been suggested as a possible mechanism, as it has in a possibly related disorder, retroperitoneal fibrosis. Inflammation in ISM has been attributed to adipocytokines, including adiponectin, resistin, leptin, IL-6, and TNF-α. Macrophages found in adipose tissue may trans-differentiate from local preadipocytes, supporting the hypothesis that adipocytes and macrophages may be interconvertible, implicating mesenteric adipose tissue in disorders such as ISM [12]. Autoimmunity has been associated with ISM, including the co-occurrence of Sjogren syndrome, sarcoidosis, rheumatoid arthritis and ankylosing spondylitis. Similarly an association of ISM and other sclero-fibrosing disorders in other organs has been reported in adults [13,14]. (More recently, ISM has been regarded as a possible subset of IgG4 related sclerosing disorders with increased expression in the lesional plasma cells. No evidence of elevated IgG4 was found in this case but the biopsy was not specifically stained for subclasses [15]. Apart from one child with SLE none of these has been reported in pediatric literature to date [16]. Our case did exhibit some features of autoimmunity with a positive ANA and hypergammaglobulinemia.

The onset of the disease may be acute, insidious or have recurring episodes before the actual diagnosis is made [17]. Varied clinical manifestations have been reported, primarily due to mechanical effects of the encasing mass/inflammation involving bowel loops, mesenteric vessels and lymphatics. These include abdominal pain with a clinically palpable mass, bilious vomiting, diarrhoea, constipation and abdominal distension. Associated features of chronic inflammation including prolonged pyrexia, weight loss, and progressive anaemia can be seen but may be more prominent in the early phase.

In the pediatric cases, of the 17 children, 4 were described as mesenteric panniculitis while the remaining 13 termed sclerosing mesenteritis. Eleven (64.7%) children presented with abdominal pain, 7 (41%) had vomiting, 4 (23.5%) with bowel symptoms in the form of constipation or diarrhoea, 8 (47%) with associated fever and 2 (11.7%) with weight loss. Only 5 (29%) children had a clinically palpable mass corroborated on exploration. Laboratory investigations were usually non specific ranging from normal to neutrophilic leucocytosis and variable elevation of acute phase reactants. Five of the 13 (38%) children tested demonstrated anemia and 9 (62%) leucocytosis. 4 (30%) children had normal white cell counts. Elevated acute phase reactants ESR/CRP were only reported in 5 children [18-26].

Most cases are diagnosed on surgical intervention, but advances in imaging techniques have helped identify features that may suggest a diagnosis of ISM. The various imaging patterns reported in children has been summarised in Table 2. Such imaging may provide valuable clues to diagnosis, clinical response and progression. Plain roentgenograms are usually non specific. However small-bowel series often show separation of loops with kinking and angulation of the small bowel, suggesting a serosal process. Folds may be thickened because of either extension of the mesenteric process into the submucosa or edema from lymphatic obstruction. The colon may occasionally be involved, with narrowing and rigidity; thumb printing also has been reported [27,28]. Ultrasonography may help delineate normal from inflammatory mesenteric fat, the latter demonstrating a homogenous echogenicity [29]. It has been reported in only 3 of the pediatric cases revealing a homogeneous low echoic mass in the involved areas. The CT appearance of sclerosing mesenteritis may vary from subtle increased attenuation in the mesentery to a solid soft-tissue mass. There may be preservation of fat around the mesenteric vessels, a phenomenon that is referred to as the "fat ring sign", a finding that may help distinguish sclerosing mesenteritis from other mesenteric processes such as lymphomas [30]. In addition, Sabate et al described the presence of a tumoral pseudocapsule in 50% of patients with mesenteric panniculitis [31]. Our case showed features of severe diffuse mesenteric adenopathy on CT. MRI usually reveals a low signal suggesting a mature fibrotic reaction. T2-weighted or fat-suppressed pulse sequences may help distinguish benign end stage fibrofatty proliferation from malignant tumours such as lymphoma [32]. Assessment of large to medium-sized vessels depicting the flow patterns and the presence of collaterals is also possible with MR imaging. MRI findings are reported in only 2 children with evidence of low signal intensities suggesting fibrosis in one case [21]. In our case, a young child, movement artefact degraded images on MR making interpretation difficult.

**Table 2:** Imaging modalities in idiopathic sclerosing mesenteritis in children

Pt no	Barium studies	CT
1	Displaced DC, Small bowel obstruction, splenomegaly	NR

2	Initial normal, then thickened folds, segmentation of contrast material.	NR

3	Normal	NR

4	Mass displacing cecum &AC.	NR

5	-------	NR

6	Mass & narrowing AC.	NR

7	NR	Heterogeneous, solid TM between spleen, left kidney and pancreas

8	NR	NR

9	Mass on medial AC	NR

10	Normal	NR

11	Narrowing of SC	Soft Tm in mid abdomen around AC to DC. Retraction of TC, Dilated AC

12	NR	NR

13	NR	NR

14	NR	Solid soft TM over the lower TC

15	NR	E/o TM over the hepatic flexure

16	Tumor mass displacing the Splenic flexure	NR

17	NR	Mesenteric adenopathy

DC; Descending colon, NR; Not reported, AC; Ascending colon, TM; Tissue mass SC; sigmoid colon, TC; Transverse colon.

Most histopathological analyses show varied degree of fibrosis, chronic inflammation, and fat necrosis with the infiltrate being predominantly lymphocytes and eosinophils with no evidence of vasculitis or vascular thickening. Foci of dystrophic calcification have been occasionally reported [5]. The findings at biopsy in the pediatric series were consistent with macroscopic changes ranging from increased adipose tissue to widespread panniculitis and extensive fibrosis (Table 3).

**Table 3:** Anatomic Involvement and pathological findings on biopsy.

Pt. no	Anatomic area of involvement	Pathology
1	Mesentery (AC and TC)	FB with LI

2	Mesentery (SB, largely at root)	Fat necrosis(early phase )→Extensive FB (later )

3	Mesentery (TI), plaques on UT, OV, PP.	FB with absent residual fat

4	T M in mesentery (TI, IC and Ap )	FB with LI and PI.

5	Mesentery (I, AC, TC, UT)	FB with LI and PI.

6	Mesentery (AC).	FB with EI.

7	TM (MG adherent to the Splenic artery)	Panniculitis and severe fibrinoid necrosis.

8	Mesentery (GC and TC )	Severe inflammatory changes minimal FB.

9	TM ( right colon and H (No obstruction ))	FB

10	Hemoperitoneum, TM (SI and TC )	FB with LI and foamy macrophages.

11	TM (Whole colon and MC (except RS) with perforation, normal SI.	FB with necrosis and LI.

12	Mesentery (IC)	Increased adipose tissue with FB with necrosis and LI.

13	TM	FB with inflammatory infiltration.

14	Mesentery (TC )	FB with inflammatory infiltration

15	Greater omentum (TC)	FB with LI and PI.

16	Mesentery (S)	FB with LI and F.

17	Extensive intraperitoneal adhesions, jejunal band with evidence of proximal obstruction	Fibro fatty changes with extensive LI and PL

AC; Ascending colon , TC; Transverse colon , FB; Fibrosis , LI; Lymphocytes infiltration , SB; Small bowel , I/TI; Ileum/Terminal Ileum, Ut; Uterus, Ov; Oviducts, TM; Tumour mass, PP; Parietal peritoneum, MG; Mesogastrium, BL; Broad ligaments, RS; Rectosigmoid colon, TM; Transverse mesocolon, S; Splenic flexure, IC; Ileocecum, GC; Gastrocolic omentum, H; Hepatic flexure EI; Eosinophils infiltration, PL; Plasma cells, F; Fibroblasts

No specific guidelines regarding the management of ISM exist and various medical and surgical modalities that have been tried. Use of anti-inflammatory, anti fibrotic and immunomodulatory agents such as cortico-steroids, methotrexate, azathioprine, colchicine, thalidomide and Tamoxifen have all been reported either alone or in combination with varied degrees of success [6,7]. Thalidomide might act by modulating the production of tumor necrosis factor-alpha (TNF-α) and other cytokines along with its inhibitory effects on angiogenesis [33]. Though reported in adults [34], our case is to our knowledge the first child to be treated with methotrexate. We used methotrexate because of the evidence of ongoing chronic inflammation and in part the reported role of methotrexate in ameliorating adiponectin which may thus inhibit the migration of macrophages to the mesenteric adipose tissue and suppress local TNF-α driven proinflammatory pathways [35].

Surgical exploration for diagnostic and therapeutic (adhesiolysis, debulking with or without resection anastomosis) purposes has been reported with all the children having some form of surgical intervention with only two cases not having actual tissue resection. Resection or debulking probably has a role in alleviating symptoms but may not help in prevention of disease progression.

## Conclusion

Widely believed to have a good outcome in adults, the paucity of reported pediatric cases and long term follow up makes it difficult to plan management and prognosticate in children.

Decisions regarding the most appropriate form and duration of therapy in children should probably be guided by the severity and chronicity of symptomatology, growth of the child, and inflammatory markers. It seems rational that unless there is severe obstruction, medical/non-surgical management might be considered. Serial imaging is probably not indicated unless warranted by a clinical deterioration. Studies in adults have reported median duration of medical treatment between 2-30 months with recurrences up to 2 years after discontinuation though long term outcomes are not well reported [6]). Acute management of the condition with corticosteroids and introduction of a steroid sparing agent such as methotrexate if treatment seems likely be required for 3 months or more could be considered a reasonable. We suggest that treatment might be continued from at least 6 months from the time clinical and laboratory remission, as for other similar chronic inflammatory conditions of childhood.

Mortality secondary to ISM has been reported in 2 children and 4 adults [16,18,33]). Chronic inflammatory diseases have a much higher morbidity in growing children and a close follow up and individualisation of care is warranted with such a potentially variable course and outcome.

## Consent

Written informed consent was obtained from the parents of the patient for publication of this case report and accompanying images. A copy of the written consent is available for review by the Editor-in-Chief of this journal.

## Competing interests

The authors declare that they have no competing interests.

## Authors' contributions

VV was involved in the review of literature, analysis and drafting the manuscript. KJM was involved in management of the case and conceptualising the design and drafting of manuscript and will act as a guarantor. Both authors read and approved the final manuscript.
